# Drought-related cholera outbreaks in Africa and the implications for climate change: a narrative review

**DOI:** 10.1080/20477724.2021.1981716

**Published:** 2021-10-02

**Authors:** Gina E. C. Charnley, Ilan Kelman, Kris A. Murray

**Affiliations:** a MRC Centre for Global Infectious Disease Analysis, Imperial College London, London, UK; b Department of Infectious Disease Epidemiology, School of Public Health, Imperial College London, London, UK; c University of Agder, Kristiansand, Norway; d Institute for Global Health, Faculty of Population Health, University College London, London, UK; e Institute for Risk and Disaster Reduction, Faculty of Mathematical and Physical Sciences, University College London, London, UK; f Mrc Unit the Gambia at London School of Hygiene and Tropical Medicine, Fajara, The Gambia

**Keywords:** *Vibrio cholerae*, outbreaks, drought, Africa, climate change

## Abstract

Africa has historically seen several periods of prolonged and extreme droughts across the continent, causing food insecurity, exacerbating social inequity and frequent mortality. A known consequence of droughts and their associated risk factors are infectious disease outbreaks, which are worsened by malnutrition, poor access to water, sanitation and hygiene and population displacement. Cholera is a potential causative agent of such outbreaks. Africa has the highest global cholera burden, several drought-prone regions and high levels of inequity. Despite this, research on cholera and drought in Africa is lacking. Here, we review available research on drought-related cholera outbreaks in Africa and identify a variety of potential mechanisms through which these outbreaks occurred, including poor access to water, marginalization of refugees and nomadic populations, expansion of informal urban settlements and demographic risks. Future climate change may alter precipitation, temperature and drought patterns, resulting in more extremes, although these changes are likely to be spatially heterogeneous. Despite high uncertainty in future drought projections, increases in drought frequency and/or durations have the potential to alter these related outbreaks into the future, potentially increasing cholera burden in the absence of countermeasures (e.g. improved sanitation infrastructure). To enable effective planning for a potentially more drought-prone Africa, inequity must be addressed, research on the health implications of drought should be enhanced, and better drought diplomacy is required to improve drought resilience under climate change.

## Introduction

*Vibrio cholerae* is a water-borne bacterial pathogen, with symptomatic cholera causing profuse watery diarrhea and sudden onset dehydration [1]. The annual cholera burden is estimated at 1.3–4 million cases and 21,000–143,000 deaths annually [2,3], with >94% of these reported in Africa [4]. Several countries are beginning to show signs of endemicity and there is currently a Global Task Force on Cholera Control that aims to reduce cholera deaths by 90% by 2030 and hopes to eliminate cholera in 20 countries [5].

Cholera outbreaks are closely related to environmental conditions with several studies exploring possible environmental and climatic links including El Niño Southern Oscillation (ENSO) [6], the Indian Ocean dipole [7] and the Intertropical Convergence Zone (ITCZ) [8]. There is also evidence for temperature and precipitation being influential in cholera outbreaks, with temperature driving epidemics and precipitation acting as a dispersal mechanism [9]. For example, a 1°C rise in temperature was associated with a 2-fold increase in cholera cases in Zanzibar [10].

Less well understood is the impact of drought on cholera, despite evidence for natural hazards resulting in disease outbreaks [11,12]. Droughts are a complex hazard and involve meteorological, hydrological, agricultural, and societal changes [13]. Africa has seen several periods of extreme and extended drought [14]. Despite some studies suggesting links between drought and cholera outbreaks [15–17], this link, especially in Africa, has been widely under-studied in isolation. One suggested mechanism through which drought may catalyze cholera outbreaks is via increasing concentrations of *V.c holerae* in groundwater. For example, in Bangladesh cholera concentrations were 13–49% higher in dry weather drainage flow samples compared to wet weather samples from floodwater [18].

Climatic and environmental factors may only be influential in cholera outbreaks to a certain threshold, after which socio-economic conditions are key drivers for outbreaks, as these increase pathogen exposure. Eight hundred and forty-four million people worldwide lack access to basic drinking water and a further 2.4 billion are without basic sanitation [5], putting many people at risk for diarrheal diseases. Human-induced factors may therefore be more important for cholera dynamics than environmental, due to a range of risk factors and potential cascades including [19], poverty [20], sanitation [21], drainage [22], water quality [23], poor healthcare [9] malnutrition [24] and human behavior such as eating practices [25], all of which could be exacerbated by drought.

Despite the risks that both drought and cholera pose in Africa and the possible links between the two, there is currently no available review that aims to collate the literature on drought-related cholera outbreaks in Africa. Here, we provide a narrative review to help bridge this knowledge gap and identify risk factors that lead to cholera outbreaks during droughts, along with identifying areas for future research. We also discuss how climate change may play a role in future droughts and cholera outbreaks, with the aim of informing drought response and resiliency plans.

## Materials & methods

We addressed two primary research questions by conducting a narrative review of the available literature, these include:

1. What are the suggested mechanisms and risk factors for drought-related cholera outbreaks in Africa?
2. How might climate change impact drought-related cholera outbreaks in Africa?

A search strategy (Table 1) was used to identify relevant literature in the following electronic databases; MEDLINE, Embase, and Global Health. Both key and MeSH (Medical Subject Heading) terms were created and varied depending on the database. Drought and cholera keywords and MeSH terms were required in combination so that at least one term from each matched. Search terms were related to [1]; cholera and [2] drought and restricted to Africa and these two terms were also used to search article titles and abstracts in Google Scholar and PubMed. The search was conducted and all papers screened in February and March 2021. Reference lists of selected papers and reviews were also screened for relevant papers. R Studio version 4.0.0 was used to manage references from the databases (package: revtools) and a web-scraping tool to extract Google Scholar search results which were then imported into R Studio.

**Table 1.** Search strategy used for MEDLINE, Embase, and GlobalHealth

Category	Keywords	MEDLINE MeSH	Embase MeSH	GlobalHealth MeSH
Drought	drought*	Droughts exp	drought exp, water stress exp	drought exp, dry conditions exp, dry season exp, water stress exp
Cholera	cholera* OR vibrio cholerae* OR outbreak*	Cholera exp, Vibrio cholerae exp, disease outbreaks exp, epidemics exp	cholera exp, Vibrio cholerae exp, epidemics exp, outbreaks exp	cholera exp, outbreaks exp, vibrio cholerae exp

No standard definitions for cholera outbreaks or droughts were set and no temporal limits were imposed, although the search was conducted in English only. Although the review was limited to studies in Africa only, specific country names were not searched to avoid missing relevant studies. A predetermined data charting form was used based on preliminary reading and the objectives of the review. Papers were screened and data extracted by the first author and included information on the publication (title, authors, date, and journal), drought details, cases, deaths, mortality rate, study area, study period, identified risk factors and methodological details. A risk factor was any factor the studies identified, either statistically or otherwise, in leading to the outbreaks or allowing transmission to proliferate. The exact wording used to describe the risk factors was extracted and risk factor terminology was then streamlined to allow for quantification of each reported risk.

## Results

A total of four studies was identified that investigated drought-related cholera outbreaks in Africa from the three databases (MEDLINE, Embase, and Global Health), out of 239 results. A further 23 studies were found using PubMed and 194,000 papers had the term drought and cholera in their title from searching Google Scholar. From these results, an additional two studies met the inclusion criteria and were included. Most studies were removed due to duplication or because they were not restricted to a country in Africa.

Full texts were available for all studies and five studies were published in English and one in French (which the search found through an English abstract). The most common methodology was case-control studies, and one paper was removed as it mentioned drought in the title but not in the main text. The studies spanned from 1974 to 2012 and were from the following countries: Mauritania [26], Mali [17], Zimbabwe [27], Mozambique [28], Cameroon [29] and Uganda [30]. A summary of the studies is shown below in Table 2.

**Table 2.** Summary of included studies

Country	Year	Study design	Cases/deaths	Reference
Mauritania	1974	Nationwide nutritional survey	149 cases, 29 deaths	[26]
Mali	1988	Case control	73 cases, 21 deaths	[17]
Zimbabwe	1996	Statistical and mathematical modeling	1,591 cases	[27]
Mozambique	1998	Descriptive epidemiology	31,000 cases, 750 deaths	[28]
Cameroon	2006	Outbreak investigation	>5,000 cases	[29]
Uganda	2012	Case control	641 cases	[30]

**Figure 1.**
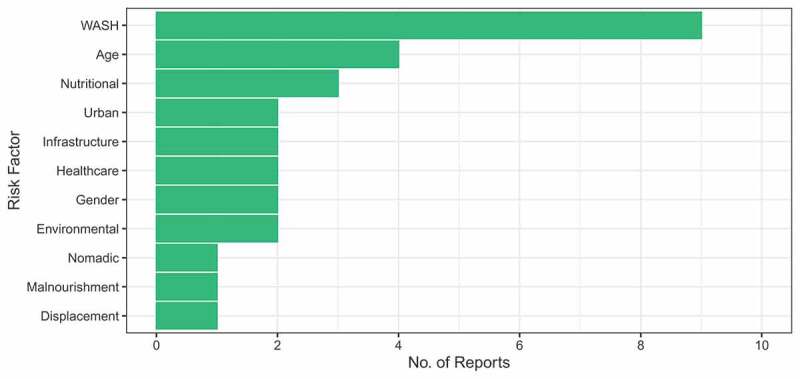
Risk factors reported in the included literature, covering those stated as being significant either statistically or not.

Several risks factors through which droughts led to cholera outbreaks were reported in the six studies and many are potentially linked in cascades. These were grouped into risk factors and presented in Figure 1. All studies mentioned more than one risk factor and some reported multiple within each risk factor group, for example, some studies reported several WASH-related risks. WASH risks were the most reported risk factor, accounting for over 30% of the total, mainly citing poor access and availability of clean water as an issue. Age-related and nutritional risk factors were also commonly reported, as young children were particularly at risk in the outbreaks and commonly suffered in drought conditions. Nutritional and malnourishment risks were separated, as nutritional risks related to specific foods (e.g. millet gruel) that were associated with the outbreak while malnourishment was related with the absence of available food.

Two studies [28,29] were purely descriptive and did not run statistical analysis on the risk factors identified. The remaining four studies all used statistical analysis to identify risk factors and only those found significant are presented in Figure 1. Odds ratios were the most common statistical analyses used and this found age to be a significant risk factor in three of the studies [17,27,30]. Other significant risk factors according to odds ratios mainly fell into the two categories below:

- Nutritional – Eating cold meals, eating road-side vendors [30], eating leftover millet gruel [17] and malnutrition [26]
- WASH – Not washing hands before eating, poor latrine access, drinking unchlorinated water, not storing water in a sealed container [30] and drinking unboiled water [26].

Alternatively, risk factors including gender and drinking locally brewed alcoholic beverages was not found significant with cholera infection in a drought setting [30].

## Discussion

### Drought-related cholera outbreaks in Africa and their associated risk factors

The work here has allowed for a more detailed understanding of drought-related cholera outbreaks and to identify which risk factors have previously been recognized. A wide range of risk factors was reported in the reviewed studies [17,26–30] and as previously suggested, human and environmental changes were important in the outbreaks [19]. Droughts can create ideal environmental conditions for *Vibrio cholerae* to proliferate and the lack of studies identified here does not necessarily account for the small number of drought-related outbreaks. An evaluation of African EM-DAT data from 1900 to 2019 found 326 droughts and 468 cholera outbreaks, with 15.2% occurring together, especially from 1970 onwards [31]. Cholera is considered a temperature sensitive pathogen [32] and has links to aquatic reservoirs [33]. Here, favorable conditions were established due to a rise in temperature, drop in rainfall, and alterations in pH and salinity, allowing algae and crustaceans to proliferate [29], which are known reservoirs for *Vibrio* species [10]. Other infectious diseases are also reported to flourish due to the conditions created by droughts including dysentery, plague [28], malaria, schistosomiasis, tuberculosis, and measles [17].

Where health systems and services are inadequate, drought is often related to human migration and displacement and has also been linked to the spread of cholera to new areas, especially along major rivers and roads [2,4,34]. Only relatively few infected individuals will show symptoms, but asymptomatic cases can still excrete the pathogen and risk contaminating the environment [20]. During the Mozambican drought of 1991–1992, an estimated 1,320,000 people were forced to seek refuge in urban areas [28], and Zimbabwe experienced a fast-moving cholera outbreak [27], after an influx of refugees from Mozambique, creating large refugee camps with typically poor conditions. A similar situation also helped catalyze a cholera outbreak in the Democratic Republic of Congo after people took refuge from the Rwandan genocide in 1994 [6].

Poor access to sanitation is a known risk for cholera outbreaks [20] and can occur after displaced populations are not provided with adequate facilities. In Zimbabwe [27] and Mali [17], poor sanitation was thought to be a main contributing factor to the cholera outbreak, due to a pit latrine density of 1/10,000 people in the refugee camps. Pit latrines though discourage open defecation, which often contaminates rivers that have multiple uses including drinker water, laundry, and bathing [30]. Camps can also impact the local population, as although residents are often prohibited from entering camps, trade between camp residents and locals is known to occur, increasing contact through food and goods [27]. Movement of people in different communities also means that there is mixed local immunity through previous infections. For example, in Douala, more than 200,000 nonimmune people are added to the local population every year, many of which live in poor conditions [29].

Further dynamics of human movement and cholera outbreaks are seen through nomadic populations who are marginalized and can be linked to poorer health outcomes during drought, possibly due to the implications on agriculture and livestock [26,30]. For example, in Mauritania, 15.5% of nomadic children compared to 8% of sedentary children fall below the threshold for severe malnutrition [26]. This is potentially due to livestock forming a large part of their diet combined with long-term forced changes undermining traditional livelihoods, such as increasing insecurity, political isolation, and an inability to access education [35]. These combined factors complicate drought relief, as nomadic populations are often harder to reach and support [17,26,30]. In Uganda, nomadic cattle herders are at heightened risk of cholera due to a combination of drought, famine and armed conflict from cross-border cattle raiding [30]. People also retain behavior when transitioning from a nomadic/rural to a sedentary/urban lifestyle that increases their risk for cholera [29,30]. For example, in Cameroon, human excreta are fed to pigs, whose waste is then used to fertilize vegetables and fruit, creating a cycle for the pathogen to sustain transmission [29].

Relying on agriculture can become tenuous during droughts, reducing food security through crop failures and livestock losses [17,28]. For example, during 1991–1992, 370,000 cattle were lost in Zimbabwe, crop production in Namibia fell by 70% and Botswana’s maize crop failed [28]. This leads to subsequent famine and malnutrition, decreasing host immune response and heightening the risk of cholera and other infectious diseases [26,36]. Drought and subsequent water scarcity lead to using different sources of food and water. For example, in Mali millet gruel is commonly eaten and acidified with curdled goat milk to prevent contamination, but in times of drought goat milk is often not available, along with several other acidifying ingredients such as lemon, tamarind, and vinegar. Famine foods are also often cooked less to preserve fuel [17]. The lack of available food increases reliance on roadside food vendors [30], which have been shown to increase cholera transmission in other outbreaks [37], often due to poor food hygiene practices, poor regulation, and no enforcement of bans.

Disruption to rural livelihoods accelerates urban expansion, forcing people into urban areas to find work. This increases unplanned urban growth which can lead to poverty and creation and expansion of informal urban settlements, a suggested risk for cholera [19,38]. Displacement to urban areas can lead to vulnerable living conditions due to unplanned urban development and sprawl, as infrastructure cannot improve in line with population growth [26–29]. Issues in urban development include a lack of health-care facilities and often an uncontrolled informal health-care sectors, which can contribute to antimicrobial resistance and increased mortality rates [29,30]. During cholera outbreaks, this can also lead to a lack of oral rehydration therapy, which can significantly increase mortality [17]. Rapid increases in urban population cause water shortages, with Douala in Cameroon suggesting that only 40% of the city’s needs were met. Low flow, poor access, and insufficient municipal services are issues, leading to septic tank dumping, stagnation, and resultant contamination [29]. This forced residents to improvise with new sources of water, which are often shared for multiple purposes, such as livestock, washing, cooking, and drinking. Wells are often un-regulated and un-protected making them vulnerable to bacterial contamination [28], while private wells dug by residents are often shallow (<1 m), making them liable to contamination [17,29].

Children [1] and women [39] are suggested demographic risk groups for cholera outbreaks. In both Zimbabwe [27] and Uganda [30], women were at a heightened risk, although in Uganda this was not found to be statistically significant. Women of child-bearing age are thought to be more at risk, due to women’s responsibility to care for sick children increasing pathogen exposure [27,30]. Other studies also identified this as a risk factor along with gender-specific eating practices, access to safe water and domestic roles [27,39]. For example, women in Sierra Leone often eat with their hands when sharing meals, as this allows them to grab more food, whereas those who eat using spoons, commonly don’t have to compete for food [39]. For children, poor hygiene practices, more vulnerable immune systems, and rapid onset dehydration can cause increased infections and mortality [40], especially in poor informal settlements [17]. In Uganda, there is also a belief that children’s feces are not infectious, increasing exposure [30]. Whereas in Mali [17], the elderly were at a heightened risk, and not a single case was reported in infants. The elderly have lower gastric acidity which potentially predisposes them to infection, while infants and babies often have some immunity to cholera due to breastfeeding.

**Figure 2.**
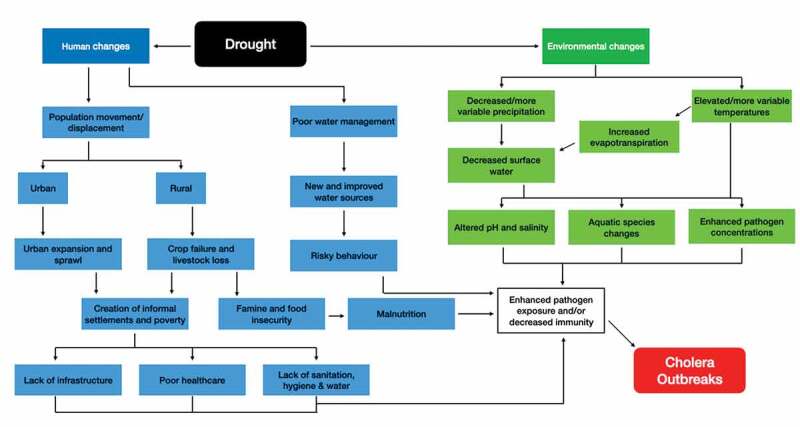
Schematic representation of potential risk factor cascades caused by droughts leading to cholera outbreaks in Africa.

Risk factors created by droughts overlap with those suggested for cholera outbreaks and here, the six studies found present how these risk factors have caused outbreaks in Africa. Below (Figure 2) is a schematic representation of the reported risk factors and how they are potentially linked to form cascades toward cholera outbreaks. The figure helps to illustrate the complexity of both the risk factors and their potential links between droughts and outbreaks. It is not considered a complete list, due to lack of available data and literature but instead some potentially important cascades.

### Implications for climate change

Several of the risk factors reported in this review and present in Figure 2, could have potential impacts on and from climate change. Spatial drought patterns and drought risk across Africa are suggested to alter with climate change [41,42]. Conversely, others suggest that droughts have not changed significantly within certain African regions historically and are not expected to under climate change on large spatial scales [43,44]. The claim that warming temperatures will lead to dryness extremes is potentially an oversimplification [45]. Although climate change may alter the hazard parameters, it will ultimately be changes in behavior that impact exposure and vulnerability, such as adaptations to alterations in water security [42,46]. One way droughts may change over certain regions of Africa is through alterations in climatic processes such as ENSO, as El Niño events lead to abnormally hot and dry conditions [28]. ENSO has already altered due to climate change, and some suggest El Niño years will become more frequent and intense with climate change [47,48], potentially impacting drought frequency and resultant cholera outbreaks.

Environmental projections can be a useful tool in highlighting how populations may be impacted by climate change and therefore for public health planning. ENSO projections using Representative Concentration Pathways emissions scenarios have shown drier conditions over east and southern Africa due to enhanced variability in rainfall [49,50]. Despite this, projections can have wide-ranging uncertainty and methodological limitations. Overall, public health and disaster risk reduction may prove far more powerful to reduce the implications of drought on cholera. For example, drought projections also suggest drier conditions in North America and Europe [51,52], but cholera is not considered a threat due to sanitation, hygiene, and access to clean drinking water. Drought forecasts can be an effective tool for cholera outbreak planning but should not be relied on, instead providing further evidence for the need to prevent disasters by reducing vulnerabilities.

Declines in precipitation have been seen over parts of Africa, while increases are seen in others, a differentiation suggested as increasing with climate change. The Indian Ocean Dipole may play a role in these changes [53,54], reducing water availability and fundamentally leading to drought in some areas. Despite this, water availability is a complex phenomenon that must account for alterations in not just the source (precipitation, groundwater, soil moisture, evapotranspiration) but also agriculture, infrastructure, and human behavior. For example, in modeling studies a net increase in freshwater resources was seen for most African countries, whereas northern regions saw more extreme dryness and serious agricultural system issues over the Sahel, Horn of Africa, and southern Africa [55]. Eighty four percentage of the population in Africa do not have access to piped water into their yard or dwelling and water fetching is mainly carried out by women and children. This domestic burden decreases the time available for education and employment, potentially stunting development if water resources become scarcer. Poor water availability is also linked to poor hygiene behaviors such as reduced hand washing, potentially increasing cholera outbreaks in these areas [43].

Urbanization and changing from a rural to urban environment found both positive and negative effects in this review. Sedentary children were found to have less malnutrition, potentially due to a wider range of and more stable food sources, along with more readily available services to urban residents. Furthermore, in Africa obesity is increasing in urban areas, especially among poorer residents. This will present new challenges in the future and potentially different health implications including non-communicable disease [56,57]. Accelerated urban expansion can result in poor living conditions and inadequate water supply, as seen in Cameroon [29]. Urbanization projections around Africa predict expansion of many urban areas, but how this will impact residents in terms of droughts affecting health will be largely determined from urban planning and responses to demographic changes, such as expanding urban water demand [58,59].

Climate change is not expected to influence everyone equally, with poorer people set to see the biggest impacts [60,61], mainly due to a lack of resources to adapt to the changing environment. In drought-stricken areas, poorer rural communities are often the worst hit, due to their reliance on agriculture and an inability to afford alternative safe water sources [62,63]. Drought can also worsen inequity, which is especially concerning for groups that are already vulnerable, such as children, the elderly, and women. Droughts can lead to alterations in revenue streams and loss of work due to illness. This is especially worrying in Africa, as some countries have large projected population growth [64], potentially putting more people at risk of extreme poverty, poor drought resilience, and subsequent cholera.

### Ways forward

Arguably the most fundamental way to reduce the impacts of drought and resultant cholera outbreaks that occur is to alleviate population vulnerabilities before the hazard occurs. This would enable people to adapt better to a changing environment, including an ability to migrate if needed and access alternative sources of food and water [65]. Population vulnerabilities highlighted here as important include enhancing the coverage of WASH in Africa, alleviating poverty and reducing the marginalization of groups, such as refugees and nomadic populations. Reducing vulnerabilities helps to diminish inequity and despite taking a significant amount of human and economic resources to accomplish [66,67], the benefits would be substantial, improving livelihoods, health and resilience to a variety of hazards, making them a cost-effective intervention.

To improve drought resilience across Africa and help to increase the capacity to cope with alterations in water availability, more awareness for the implications of drought on health and disease is needed, by enhancing research and technology. This will enable surveillance and forecasting and allow prioritization of areas for outbreak prevention. Health and ecological monitoring need to be more integrated [28], assessing health under an interdisciplinary lens, as a better understanding of the connections between the environment and disease emergence can promote broader awareness. Groundwater availability maps could be one method of doing this by improving the understanding of vulnerability through water and food security-insecurity zones. Data collected also needs to be shared to allow for further work and better collaboration [67,68].

Drought diplomacy needs to be improved in many areas, with multi-national agreements for sharing and managing rivers and other waterways [28]. This will improve the capacity to cope with drought and foster functional relationships that may allow for future co-operation [69]. It should aim to prevent the nationalization of water sources, as how one country manages a water source can have a knock-on effect and droughts rarely affect a single country in isolation. Greater government response and plans are needed as drought assistance is largely by humanitarian aid organizations and more work is needed to make countries more self-sufficient and to support disaster prevention alongside rapid and targeted responses. For example, in southern Africa, the Horn of Africa and Ethiopian droughts, people were left without access to water, leading to outbreaks of disease including cholera, while non-food assistance including water interventions have been largely ineffective at addressing immediate needs [63].

When outbreaks do occur, responses need to be rapid, due to the short incubation period of cholera (2 hours – 5 days). For example, in Mali, most cases and deaths were reported in the first week of the outbreak [17]. In Mauritania, the cholera outbreak was reported almost immediately and closure of contaminated wells, intensive vaccination, chemoprophylaxis for contacts and education campaigns meant that the outbreak was terminated within 3 weeks [26]. Despite this, chemoprophylaxis should be targeted, as not everyone is at risk, and it can lead to accelerated resistance [17]. Global policy on these actions may help those involved in the response to move swiftly during drought and stop outbreaks in the early stages, alongside rapid assessment of a population during and after a natural hazard to prioritize areas for aid and food distribution [26].

### Limitations

One of the main limitations of this study is the small sample size and varying methodologies, making conclusions based on such limited literature challenging and a quantitative analysis of the risk factors not robust. The most recent study found through the search strategy is currently 9 years since publication; therefore, recommendations made in these studies may be outdated. Methodological issues include a lack of environmental analysis in the reviewed studies, represented on the right-hand of Figure 2. Improvements are a potential priority when investigating the infectious disease risks in future droughts. Furthermore, risk factor analysis is often subjective and risk factors identified as important will depend on the questions that were asked during the studies and the questionnaire design.

Cholera is known to be widely under-reported globally, with only around 5–10% of cases thought to be reported to the World Health Organization [70]. This is further complicated by a high percentage of asymptomatic cases; therefore, large serological surveys are needed to understand the proportion of cases that show symptoms [27]. This also creates issues when trying to understand mortality rates and several drought-related cases and outbreaks may be missed. For example, in Mali [17], high case fatality was thought to be due to only severe cases being reported.

Another issue is how drought has been potentially quantified in the studies used here. Deciding when a drought begins and ends and then assigning this to a health outcome is challenging and it is not possible to derive a single definition for drought, as what constitutes a drought in one area is different from another [71]. This may account for why there are few multi-national drought response plans.

## Conclusion

This review has helped to illustrate the current understanding and mechanisms for drought-related cholera outbreaks in Africa. To the authors’ knowledge this is the first review to collate this information and hopefully will facilitate more drought and disease research, which is a chronically understudied area. Drought heightens inequity, making the perfect conditions for infectious diseases to spread, and here examples are provided of how this can occur in relation to cholera, through alteration in both the environment and human behavior. Figure 2 shows the large variety of human behavior that is cited as important in these outbreaks. It provides further evidence that these socio-economic conditions are potentially more important for cholera outbreaks than the environment, as these enable a human-environment-pathogen link to be made. Despite a lack of consensus on future drought changes in Africa with climate change, there does appear to be an agreement on an increased variability in climate extremes, with spatially heterogeneous drought changes. This needs to be at the forefront of future drought planning to make sure actions remain effective under climate change.

The work here stresses the need for reducing population vulnerability before hazards occur. This could reduce drought impacts in Africa especially through poverty alleviation and providing WASH services, as the benefits of this would be far-reaching. Technological improvements, increasing research, and applying the research results to tackling drought-related health impacts is necessary. Additionally, enhanced drought diplomacy-that is, calling for multi-country drought response plans and water agreements-would allow for better water management and resource sharing and rapid assistance during droughts and subsequent outbreaks. This must include real-time assessments of population needs and identification of risk areas and local inequity, to make sure that assistance is targeted. With this in place, the risk factors created by drought can be reduced and therefore the number of drought-related cholera outbreaks minimized, ultimately reducing morbidity and mortality and alleviating the burden of cholera in Africa.
